# Supplementary material for: A randomized clinical trial assessing the effect of automated medication-targeted alerts on acute kidney injury outcomes
Source: Nat Commun. 2023 May 17;14:2826. doi: 10.1038/s41467-023-38532-3 (PMC10192367; doi:10.1038/s41467-023-38532-3)
Supplement: Supplementary file 1 — Supplementary Information [file 41467_2023_38532_MOESM1_ESM.pdf]

Supplemental Table 1

PPI	NSAID	ACE/ARB
dexlansoprazole	celecoxib	aliskiren
esomeprazole	diclofenac	azilsartan
lansoprazole	etodolac	benazepril
omeprazole	flurbiprofen	candesartan
pantoprazole	ibuprofen	captopril
raberprazole	indomethacin	enalapril
	ketoprofen	eprosartan
	ketorolac	fosinopril
	meloxicam	ibersartan
	naproxen	lisinopril
	piroxicam	losartan
	sulindac	moexipril
		olmesartan
		perindopril
		quinapril
		ramipril
		telmisartan
		trandolapril
		valsartan

Supplemental Table 1: Individual medication classifications. Note these reflect medications available on the study hospitals' inpatient formularies at the time of the trial, not all medications in the class. Only oral and IV routes were considered as exposures (eg ophthalmic ketorolac would not trigger an alert).

Supplemental Table 2: Details of Study Hospitals

Hospital	Location	Beds	Number of admissions during the study period	Number (%) of admissions enrolled in ELAIA-2
Hospital 1	New Haven, CT	1030	55,923	2447 (4.4%)
Hospital 2	New Haven, CT	511	17,329	1214 (7.0%)
Hospital 3	Bridgeport, CT	383	24,318	910 (3.7%)
Hospital 4	Greenwich, CT	206	17,163	489 (2.8%)

Supplemental Table 2: Study hospitals

Supplemental Table 3 – Missing Data

**Covariate**	**Missing in (number, %)**
Baseline anion gap	29 (0.6%)
Baseline serum bicarbonate	24 (0.5%)
Baseline BUN	26 (0.6%)
Baseline chloride	26 (0.5%)
Baseline hemoglobin	36 (0.7%)
Baseline platelet count	44 (0.9%)
Baseline potassium	29 (0.6%)
Baseline sodium	26 (0.5%)
Baseline white blood cell count	43 (0.9%)

Supplemental Table 3 – Missing Data. All outcome data was complete for all enrolled participants. Counts of missing baseline covariates are reported here.

Supplemental Table 4: Baseline characteristics, NSAID Subgroup

**Characteristic**	**Alert (N=748)**	**Usual Care (N=805)**
Demographics		
Age (years)	62 (50,73)	63 (53,74)
Female sex	397 (53%)	442 (55%)
Black	170 (23%)	173 (21%)
Hispanic	139 (19%)	125 (16%)
Hospital Location		
Medical admission	516 (69%)	573 (71%)
Patient in the ICU	151 (20%)	172 (21%)
Patient in the emergency department	36 (5%)	28 (3%)
Patient in the ward	535 (72%)	573 (71%)
Hospital 1	344 (46%)	399 (50%)
Hospital 2	187 (25%)	208 (26%)
Hospital 3	141 (19%)	141 (18%)
Hospital 4	76 (10%)	57 (7%)
Comorbidities		
Chronic kidney disease	111 (15%)	115 (14%)
Congestive heart failure	170 (23%)	181 (22%)
COPD	232 (31%)	253 (31%)

Diabetes mellitus	247 (33%)	260 (32%)
Hypertension	456 (61%)	513 (64%)
Malignancy	142 (19%)	204 (25%)
Depression	204 (27%)	232 (29%)
Liver disease	94 (13%)	115 (14%)
AKI stage at randomization		
Stage 0*	2 (0%)	0 (0%)
Stage 1	651 (87%)	680 (84%)
Stage 2	72 (10%)	104 (13%)
Stage 3	23 (3%)	21 (3%)
Laboratory Values		
eGFR on admission (ml/min/1.73 m^2)	79.1 (53.4,99.7)	78.6 (53.4,99.8)
Creatinine at randomization (mg/dL)	1.3 (1,1.7)	1.3 (1,1.6)
Creatinine at admission (mg/dL)	0.9 (0.7,1.3)	1 (0.7,1.3)
Sodium (meq/L)	137 (134,140)	138 (135,140)
Potassium (meq/L)	4.2 (3.8,4.5)	4.1 (3.7,4.5)
Chloride (meq/L)	102 (99,106)	102.5 (99,106)
Bicarbonate (meq/L)	23 (21,26)	23 (21,26)
Anion gap (meq/L)	11 (9,13)	11 (10,14)
Blood urea nitrogen (mg/dL)	23 (16,35)	24 (16,35)

White blood cell count (x1000/uL)	10 (7.1,14)	9.7 (6.7,14)
Hemoglobin (g/dL)	10.7 (9,12.5)	10.8 (9.1,12.5)
Platelet count (x1000/uL)	220 (164.5,298.5)	228 (155,304)
Modified SOFA Score	2 (1,3)	2 (1,4)
Exposures prior to AKI		
Contrast in prior 72 hours	198 (26%)	206 (26%)
RAASi	320 (43%)	369 (46%)
NSAID	748 (100%)	805 (100%)
PPI	429 (57%)	459 (57%)
One medication of Interest	157 (21%)	150 (19%)
Two medications of interest	433 (58%)	482 (60%)
Three medications of interest	158 (21%)	173 (21%)

Supplemental Table 5: Baseline characteristics, RAASi Subgroup

**Characteristic**	**Alert (N=1350)**	**Usual Care (N=1329)**
Demographics		
Age (years)	72 (62,82)	73 (62,81)
Female sex	637 (47%)	658 (50%)
Black	288 (21%)	281 (21%)
Hispanic	187 (14%)	165 (12%)

Hospital Location		
Medical admission	1021 (76%)	1023 (77%)
Patient in the ICU	244 (18%)	235 (18%)
Patient in the emergency department	49 (4%)	56 (4%)
Patient in the ward	1013 (75%)	1002 (75%)
Hospital 1	609 (45%)	577 (43%)
Hospital 2	362 (27%)	369 (28%)
Hospital 3	247 (18%)	243 (18%)
Hospital 4	132 (10%)	140 (11%)
Comorbidities		
Chronic kidney disease	385 (29%)	359 (27%)
Congestive heart failure	496 (37%)	471 (35%)
COPD	420 (31%)	417 (31%)
Diabetes mellitus	622 (46%)	600 (45%)
Hypertension	1048 (78%)	1067 (80%)
Malignancy	249 (18%)	266 (20%)
Depression	306 (23%)	293 (22%)
Liver disease	110 (8%)	120 (9%)
AKI stage at randomization		
Stage 0*	4 (0%)	2 (0%)
Stage 1	1239 (92%)	1191 (90%)
Stage 2	90 (7%)	113 (9%)

Stage 3	17 (1%)	23 (2%)
Laboratory Values		
eGFR on admission (ml/min/1.73 m^2)	58 (38.6,81.3)	57.4 (38.4,79.7)
Creatinine at randomization (mg/dL)	1.5 (1.2,1.9)	1.5 (1.2,2)
Creatinine at admission (mg/dL)	1.2 (0.9,1.6)	1.2 (0.9,1.6)
Sodium (meq/L)	138 (135,140)	138 (135,140)
Potassium (meq/L)	4.2 (3.9,4.6)	4.2 (3.8,4.6)
Chloride (meq/L)	102 (99,105)	102 (99,105)
Bicarbonate (meq/L)	24 (21,26)	24 (21,26)
Anion gap (meq/L)	12 (10,14)	12 (10,14)
Blood urea nitrogen (mg/dL)	30 (22,42)	30 (22,42)
White blood cell count (x1000/uL)	9.2 (6.9,12.7)	9.3 (6.5,12.7)
Hemoglobin (g/dL)	11.1 (9.5,12.9)	11.1 (9.5,12.8)
Platelet count (x1000/uL)	222 (168,291)	225 (168,290.5)
Modified SOFA Score	2 (1,3)	2 (1,3)
Exposures prior to AKI		
Contrast in prior 72 hours	307 (23%)	326 (25%)
RAASi	1350 (100%)	1329 (100%)
NSAID	320 (24%)	369 (28%)
PPI	629 (47%)	595 (45%)

One medication of Interest	559 (41%)	538 (40%)
Two medications of interest	633 (47%)	618 (47%)
Three medications of interest	158 (12%)	173 (13%)

Supplemental Table 6: Baseline charactersitics, PPI Subgroup

**Characteristic**	**Alert (N=1654)**	**Usual Care (N=1644)**
Demographics		
Age (years)	71 (59,81)	70 (59,80)
Female sex	809 (49%)	766 (47%)
Black	306 (19%)	268 (16%)
Hispanic	224 (14%)	230 (14%)
Hospital Location		
Medical admission	1284 (78%)	1284 (78%)
Patient in the ICU	435 (26%)	473 (29%)
Patient in the emergency department	58 (4%)	48 (3%)
Patient in the ward	1078 (65%)	1051 (64%)
Hospital 1	793 (48%)	868 (53%)
Hospital 2	374 (23%)	336 (20%)
Hospital 3	323 (20%)	300 (18%)
Hospital 4	164 (10%)	140 (9%)
Comorbidities		

Chronic kidney disease	457 (28%)	423 (26%)
Congestive heart failure	563 (34%)	541 (33%)
COPD	553 (33%)	518 (32%)
Diabetes mellitus	618 (37%)	579 (35%)
Hypertension	1093 (66%)	1101 (67%)
Malignancy	409 (25%)	402 (24%)
Depression	419 (25%)	411 (25%)
Liver disease	250 (15%)	292 (18%)
AKI stage at randomization		
Stage 0*	4 (0%)	2 (0%)
Stage 1	1486 (90%)	1456 (89%)
Stage 2	124 (7%)	153 (9%)
Stage 3	40 (2%)	33 (2%)
Laboratory Values		
eGFR on admission (ml/min/1.73 m^2)	56.3 (36.1,84.6)	59.2 (37.2,87.6)
Creatinine at randomization (mg/dL)	1.5 (1.2,2)	1.5 (1.2,2)
Creatinine at admission (mg/dL)	1.2 (0.9,1.7)	1.2 (0.9,1.7)
Sodium (meq/L)	138 (135,141)	138 (135,141)
Potassium (meq/L)	4.2 (3.8,4.6)	4.2 (3.8,4.6)
Chloride (meq/L)	102 (98,106)	102 (99,107)
Bicarbonate (meq/L)	23 (20,26)	23 (20,26)

Anion gap (meq/L)	12 (10,14)	12 (10,14)
Blood urea nitrogen (mg/dL)	30 (20,44)	30 (20,43)
White blood cell count (x1000/uL)	9.7 (6.6,14)	9.8 (6.6,14.3)
Hemoglobin (g/dL)	10.1 (8.5,12.1)	10.1 (8.5,12)
Platelet count (x1000/uL)	206 (141,286)	206 (134,284)
Modified SOFA Score	3 (1,4)	3 (2,4)
Exposures prior to AKI		
Contrast in prior 72 hours	432 (26%)	460 (28%)
RAASi	629 (38%)	595 (36%)
NSAID	429 (26%)	459 (28%)
PPI	1654 (100%)	1644 (100%)
One medication of Interest	754 (46%)	763 (46%)
Two medications of interest	742 (45%)	708 (43%)
Three medications of interest	158 (10%)	173 (11%)

Supplemental Table 7: Mediation analysis investigating whether the effect of electronic health record alerts on the composite outcome is mediated by cessation of PPI among the PPI subgroup.

	Natural Indirect Effect[a] (95% CI)	Total Effect[a] (95% CI)	Proportion Mediated[b] (95% CI)
Unadjusted Analysis[c]	0.979 (0.963, 0.993)	0.823 (0.715, 0.967)	10.7% (2.9%, 44.7%)
Adjusted Analysis[d]	0.983 (0.967,0.994)	0.829 (0.705, 0.964)	9.0% (2.1%, 39.2%)

[a] Natural indirect effect and total effect were calculated on an odds ratio scale.

[b] Proportion mediated = log(natural indirect effect)/log(total effect)×100%

[c] not adjust for any pre-treatment covariates.

[d] adjusted for age, sex, ICU at randomization, Creatinine, hemoglobin, plateletcount, and time from admission to randomization. The natural indirect effect and proportion mediated in the adjusted analysis were reported for the mean level of those covariates.

Supplemental Table 8: Secondary Outcomes, by MOI Subgroup

Outcome			
NSAID Subgroup	**Alert (N=748)**	**Usual Care (N=805)**	**Relative Risk (95% CI)**
Progression of AKI	131 (17.5%)	147 (18.3%)	0.96 (0.78 to 1.19)
Dialysis	25 (3.3%)	32 (4%)	0.83 (0.49 to 1.4)
Death	52 (7%)	74 (9.2%)	0.75 (0.54 to 1.05)
Inpatient Mortality	76 (10.2%)	89 (11.1%)	0.91 (0.68 to 1.21)
Inpatient Dialysis	28 (3.7%)	35 (4.3%)	0.86 (0.53 to 1.4)
Progression to stage 2 AKI	71 (9.5%)	71 (8.8%)	1.08 (0.79 to 1.48)
Progression to stage 3 AKI	60 (8%)	76 (9.4%)	0.85 (0.61 to 1.18)
30-day readmission	95 (12.7%)	115 (14.3%)	0.91 (0.71 to 1.17)
Inpatient renal consult (post randomization)	80 (10.7%)	92 (11.4%)	0.96 (0.73 to 1.28)
Duration of AKI	1 (0.7,2)	1 (0.7,2)	p-value: 0.16
LOS since alert	4.4 (2.1,10.1)	4.4 (2.1,10.3)	p-value: 0.85
RAASi Subgroup	**Alert (N=1350)**	**Usual Care (N=1329)**	**Relative Risk (95% CI)**
Progression of AKI	220 (16.3%)	208 (15.7%)	1.03 (0.87 to 1.23)
Dialysis	46 (3.4%)	42 (3.2%)	1.07 (0.71 to 1.61)
Death	90 (6.7%)	98 (7.4%)	0.9 (0.69 to 1.19)
Inpatient Mortality	132 (9.8%)	129 (9.7%)	1 (0.8 to 1.26)
Inpatient Dialysis	58 (4.3%)	46 (3.5%)	1.23 (0.84 to 1.8)
Progression to stage 2 AKI	121 (9%)	117 (8.8%)	1.01 (0.79 to 1.29)
Progression to stage 3 AKI	97 (7.2%)	91 (6.8%)	1.04 (0.79 to 1.37)
30-day readmission	160 (11.9%)	188 (14.1%)	0.84 (0.69 to 1.02)
Inpatient renal consult (post randomization)	181 (13.4%)	155 (11.7%)	1.14 (0.93 to 1.39)
Duration of AKI	1 (0.8,2)	1.1 (0.8,2)	p-value: 0.13
LOS since alert	4.7 (2.1,10.4)	4.4 (2.2,9.8)	p-value: 0.28
PPI Subgroup	**Alert (N=1654)**	**Usual Care (N=1644)**	**Relative Risk (95% CI)**
Progression of AKI	349 (21.1%)	399 (24.3%)	0.88 (0.78 to 1)
Dialysis	101 (6.1%)	114 (6.9%)	0.91 (0.7 to 1.18)
Death	215 (13%)	237 (14.4%)	0.9 (0.76 to 1.07)
Inpatient Mortality	300 (18.1%)	306 (18.6%)	0.98 (0.85 to 1.13)
Inpatient Dialysis	124 (7.5%)	119 (7.2%)	1.08 (0.85 to 1.37)
Progression to stage 2 AKI	168 (10.2%)	181 (11%)	0.93 (0.76 to 1.13)
Progression to stage 3 AKI	180 (10.9%)	217 (13.2%)	0.84 (0.7 to 1.01)
30-day readmission	227 (13.7%)	227 (13.8%)	1 (0.85 to 1.19)

Inpatient renal consult (post randomization)	252 (15.2%)	287 (17.5%)	0.89 (0.77 to 1.04)
Duration of AKI	1.1 (0.8,2.3)	1.1 (0.8,2.8)	p-value : .12
LOS since alert	6 (2.4,13.1)	6 (2.4,12.4)	p-value: .68

Supplemental Table 8: Secondary outcomes among those exposed to various medications of interest at at the time of randomization.

Supplemental Figure 1: Overlap of MOIs

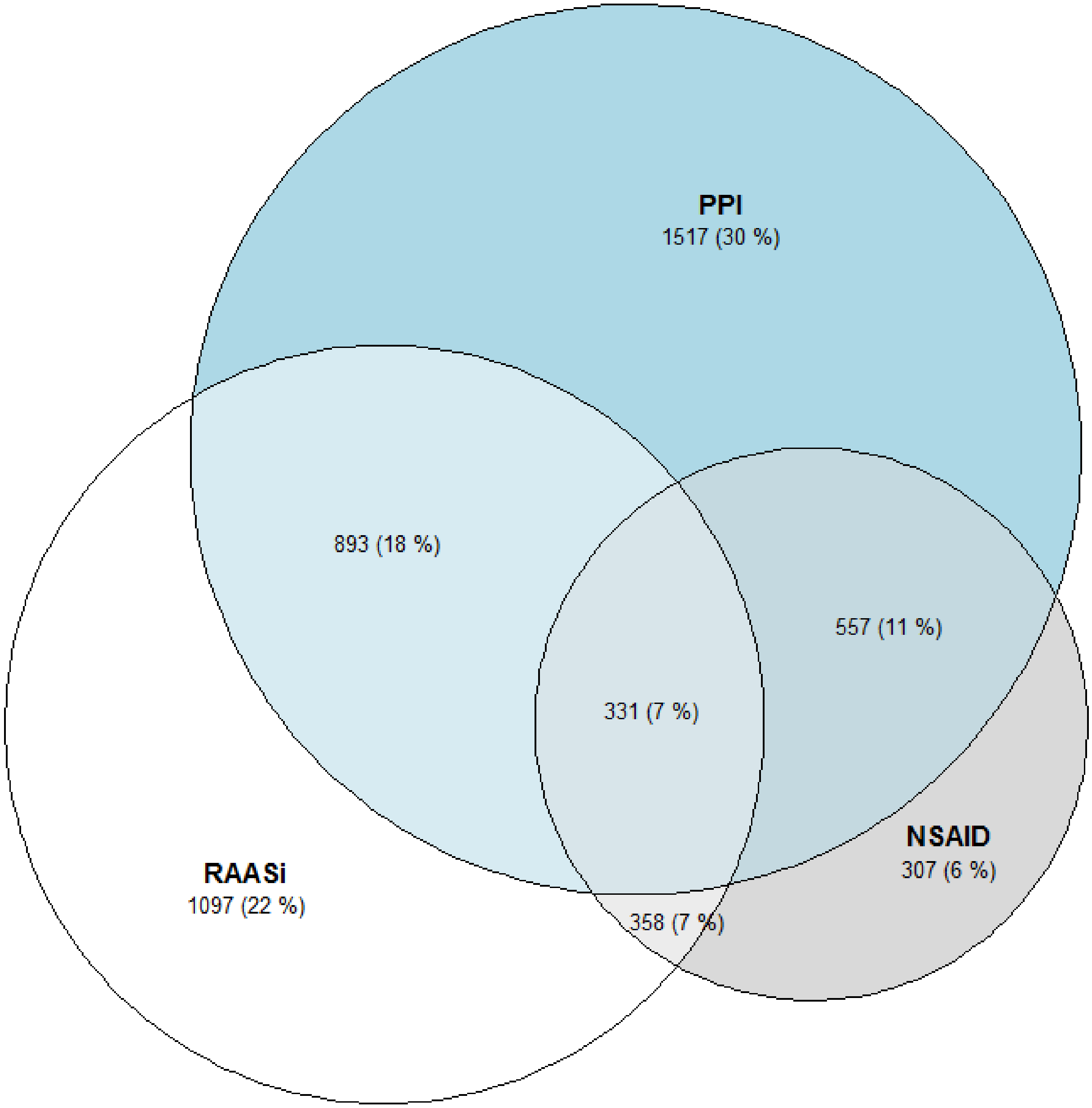

Supplemental Figure 1: Overlap of medications of interest. Venn diagram showing the frequency with which multiple MOIs were ordered at the time of randomization.

Supplemental Figure 2A: Effect of Alerts on MOI Discontinuation Over Study Time

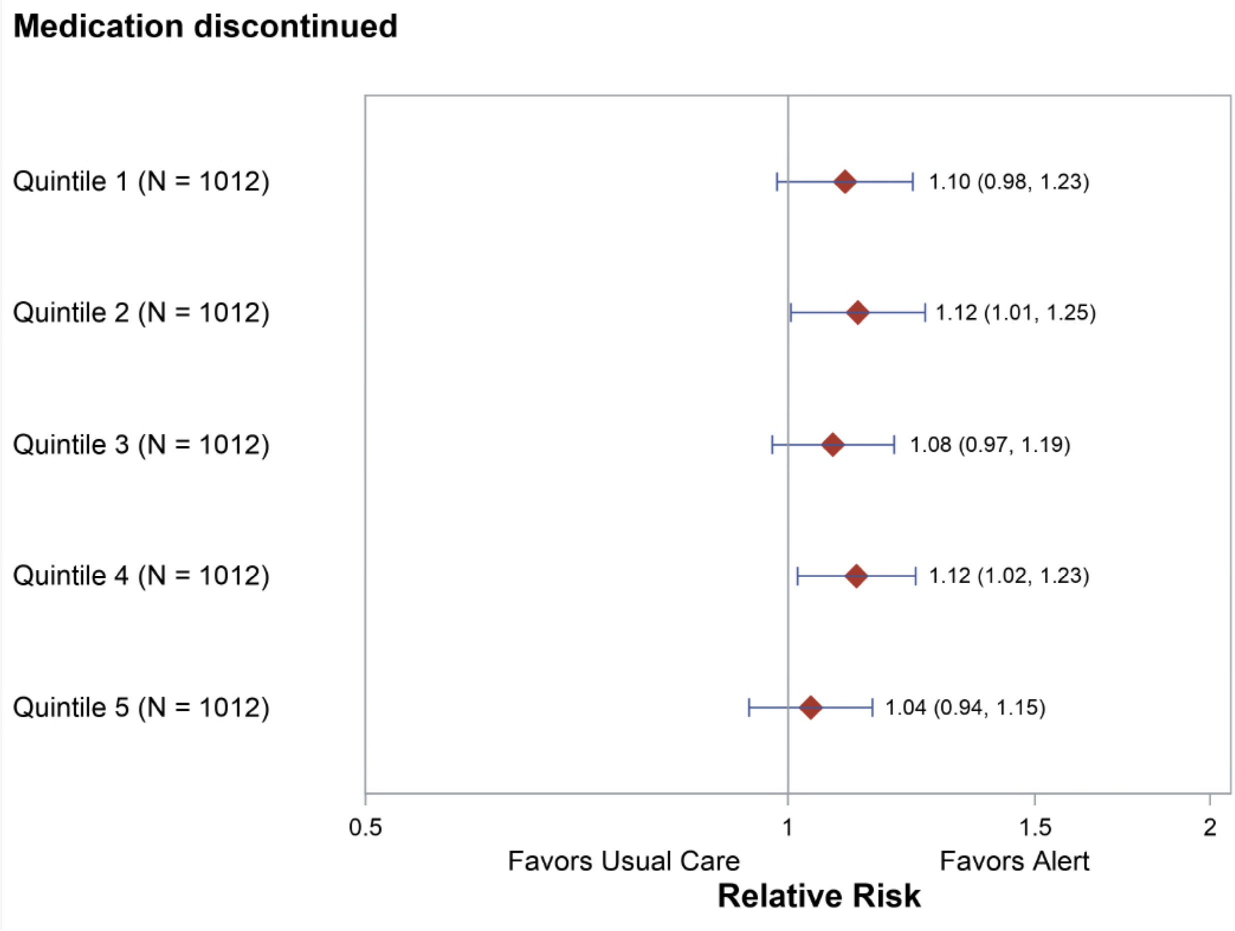

Supplemental Figure 2A: Effect of alerts on MOI discontinuation over study time. Quintiles represent the first 20% of participants enrolled, the next 20%, and so on. Significant contamination would present as an attenuation of alert effect as more patients are enrolled (giving providers more opportunity to "learn" the intervention). No such effect was seen (p-for interaction trend=0.79). Error bars represent 95% confidence intervals. Source data are provided as a Source Data file.

Supplemental Figure 2B: Effect of alerts on primary outcome over study time

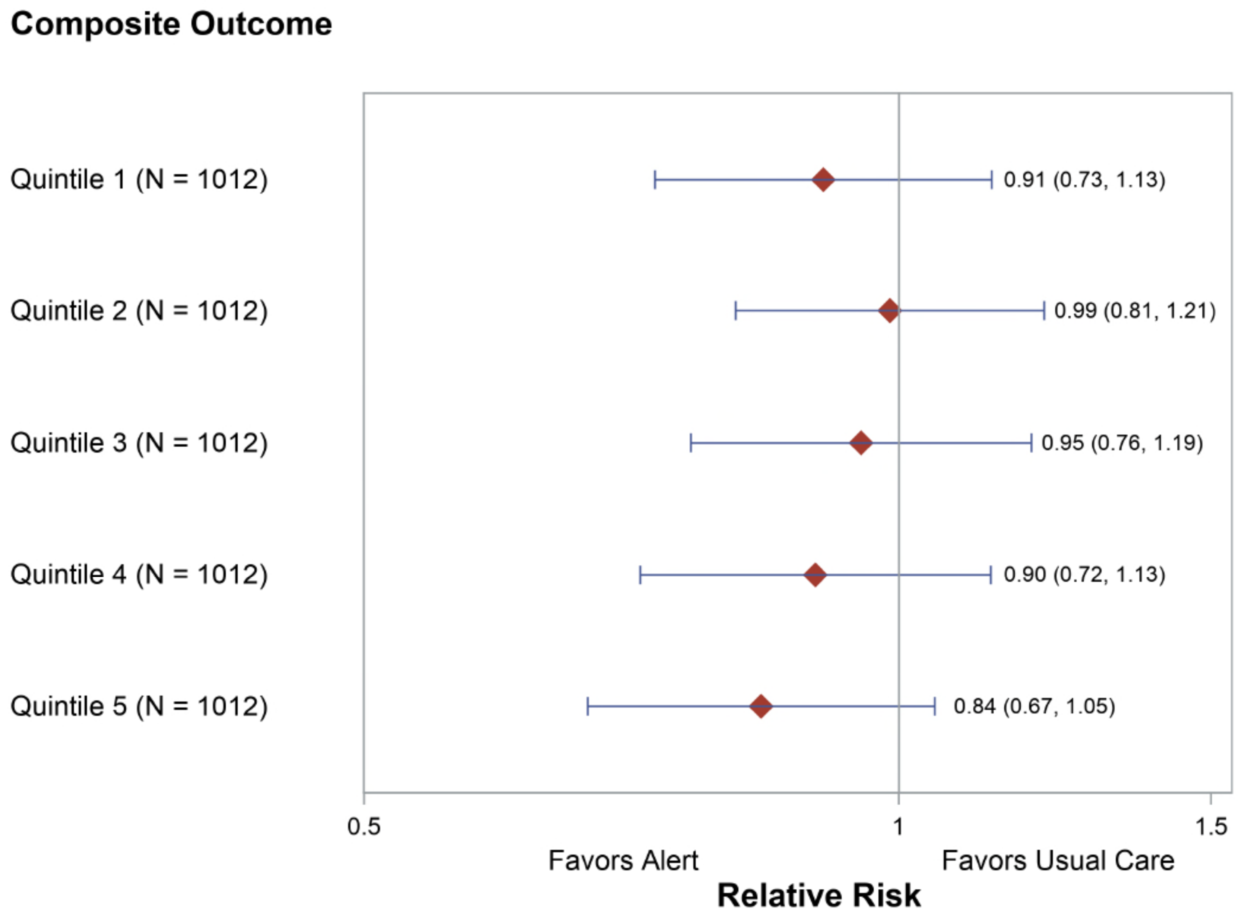

Supplemental Figure 2B: Effect of alerts on primary outcome over study time.

Quintiles represent the first 20% of participants enrolled, the next 20%, and so on. Significant contamination would present as an attenuation of alert effect as more patients are enrolled (giving providers more opportunity to "learn" the intervention). No such effect was seen (p-for interaction trend=0.51). Error bars represent 95% confidence intervals. Source data are provided as a Source Data file.

Supplementary Note 1

CONSORT 2010 checklist of information to include when reporting a randomised trial*

Section/Topic	Item No	Checklist item	Reported on page No
**Title and abstract**			
	1a	Identification as a randomised trial in the title	1
	1b	Structured summary of trial design, methods, results, and conclusions (for specific guidance see CONSORT for abstracts)	2
**Introduction**			
Background and objectives	2a	Scientific background and explanation of rationale	3-4
	2b	Specific objectives or hypotheses	4
**Methods**			
Trial design	3a	Description of trial design (such as parallel, factorial) including allocation ratio	12
	3b	Important changes to methods after trial commencement (such as eligibility criteria), with reasons	N/A
Participants	4a	Eligibility criteria for participants	12-13
	4b	Settings and locations where the data were collected	13; Supp Table 2
Interventions	5	The interventions for each group with sufficient details to allow replication, including how and when they were actually administered	13-14
Outcomes	6a	Completely defined pre-specified primary and secondary outcome measures, including how and when they were assessed	14-16
	6b	Any changes to trial outcomes after the trial commenced, with reasons	N/A
Sample size	7a	How sample size was determined	15
	7b	When applicable, explanation of any interim analyses and stopping guidelines	15-16
Randomisation:			
Sequence generation	8a	Method used to generate the random allocation sequence	13
	8b	Type of randomisation; details of any restriction (such as blocking and block size)	13
Allocation concealment mechanism	9	Mechanism used to implement the random allocation sequence (such as sequentially numbered containers), describing any steps taken to conceal the sequence until interventions were assigned	13
Implementation	10	Who generated the random allocation sequence, who enrolled participants, and who assigned participants to interventions	13
Blinding	11a	If done, who was blinded after assignment to interventions (for example, participants, care providers, those	13

		assessing outcomes) and how	
	11b	If relevant, description of the similarity of interventions	14
Statistical methods	12a	Statistical methods used to compare groups for primary and secondary outcomes	16
	12b	Methods for additional analyses, such as subgroup analyses and adjusted analyses	16
**Results**			
Participant flow (a diagram is strongly recommended)	13a	For each group, the numbers of participants who were randomly assigned, received intended treatment, and were analysed for the primary outcome	12; figure 1
	13b	For each group, losses and exclusions after randomisation, together with reasons	N/A
Recruitment	14a	Dates defining the periods of recruitment and follow-up	4
	14b	Why the trial ended or was stopped	N/A
Baseline data	15	A table showing baseline demographic and clinical characteristics for each group	23-26: table 1
Numbers analysed	16	For each group, number of participants (denominator) included in each analysis and whether the analysis was by original assigned groups	Figure 1
Outcomes and estimation	17a	For each primary and secondary outcome, results for each group, and the estimated effect size and its precision (such as 95% confidence interval)	5-6; figure 3, table 2
	17b	For binary outcomes, presentation of both absolute and relative effect sizes is recommended	Table 2
Ancillary analyses	18	Results of any other analyses performed, including subgroup analyses and adjusted analyses, distinguishing pre-specified from exploratory	5-7
Harms	19	All important harms or unintended effects in each group (for specific guidance see CONSORT for harms)	7; table 3
**Discussion**			
Limitations	20	Trial limitations, addressing sources of potential bias, imprecision, and, if relevant, multiplicity of analyses	10-11
Generalisability	21	Generalisability (external validity, applicability) of the trial findings	11
Interpretation	22	Interpretation consistent with results, balancing benefits and harms, and considering other relevant evidence	8-10
**Other information**			
Registration	23	Registration number and name of trial registry	2
Protocol	24	Where the full trial protocol can be accessed, if available	Included with submission
Funding	25	Sources of funding and other support (such as supply of drugs), role of funders	4, 21

*We strongly recommend reading this statement in conjunction with the CONSORT 2010 Explanation and Elaboration for important clarifications on all the items. If relevant, we also recommend reading CONSORT extensions for cluster randomised trials, non-inferiority and equivalence trials, non-pharmacological treatments, herbal interventions, and pragmatic trials. Additional extensions are forthcoming: for those and for up to date references relevant to this checklist, see www.consort-statement.org.

Supplementary Note 2

HRP-503B – BIOMEDICAL RESEARCH PROTOCOL
(2017-1)

Protocol Title: Electronic Alerts for Acute Kidney Injury Amelioration (ELAIA-2): A Multi-Center, Randomized, Controlled Trial

Principal Investigator: F. Perry Wilson, MD MSCE

Version Date: July 10th, 2020

(*If applicable*) Clinicaltrials.gov Registration #: NCT02771977

INSTRUCTIONS
This template is intended to help investigators prepare a protocol that includes all of the necessary information needed by the IRB to determine whether a study meets approval criteria. **Read the following instructions before proceeding:**<br><br>1. Use this protocol template for a PI initiated study that includes direct interactions with research subjects. Additional templates for other types of research protocols are available in the system Library.<br>2. If a section or question does not apply to your research study, type "Not Applicable" underneath.<br>3. Once completed, upload your protocol in the "Basic Information" screen in IRES IRB system.

Section I: Research Plan

1. **Statement of Purpose:**
The purpose of this study is to assess the efficacy of medication-targeted AKI alerts in improving the rate of cessation of nephrotoxic agents within 24 hours of AKI onset, and to determine if such alerts will reduce the rates of AKI progression, dialysis and death in hospitalized patients, followed for up to 14 days past randomization.

2. **Probable Duration of Project:**
We expect to complete patient enrollment within 24 months, and to conduct analysis and publishing activities within the following 6 months.

3. **Background:**
Acute kidney injury (AKI) carries a significant, independent risk of mortality among hospitalized patients.[1,2] A common cause of AKI in hospitalized patients is exposure to nephrotoxic agents and medications, with multiple studies showing strong associations between NSAIDs and RAAS agents with worsening renal function and AKI. [3-7] However, the true impact of certain agents on the progression of AKI remains unclear.
This study will focus on three commonly prescribed nephrotoxic agents. Non-steroidal anti-inflammatory drugs, or NSAIDs, inhibit cyclooxygenase-dependent prostacyclin formation. This can result in suppressed vasodilation that would otherwise preserve glomerular filtration rate (GFR) in situations of decreased circulating volume. [8] Consequently, recommendations by the Kidney Disease: Improving Global Outcomes (KDIGO) consortium state that, in the setting of AKI, "concurrent nephrotoxic medication – including, in particular, NSAIDs…should be preferably stopped". [9] RAAS agents decrease the GFR by decreasing hydrostatic pressure in the glomerulus and can lead to a 30% increase in creatinine in the absence of AKI. Conversely, these agents increase renal blood flow, which may prove beneficial in the setting of AKI by preventing ischemic damage in the kidney. [10,11] Lastly, proton-pump inhibitors, or PPIs, have also been linked to acute kidney injury. Evidence for a link between PPI use and Acute Interstitial Nephritis (AIN) is quite strong, despite being observational. [12,13] In a study of nearly 600,000 older residents of Ontario, Antoniou et al found that the use of PPI was associated with a hazard ratio for AKI of 2.52 (2.27 – 2.79). [14] Only 2% of the AKI cases were attributable to AIN, suggesting the potential for direct nephrotoxic effects, though the study design could not account for residual confounding. Recent studies (including one co-authored by the PI of this proposal) have also linked PPI use to chronic kidney disease. [15,16]
Despite international guidelines for the treatment of AKI that focus on appropriate drug dosing and avoidance of nephrotoxic exposures, multiple studies have demonstrated that appropriate diagnostic and therapeutic interventions are inconsistently performed. [9,17-21] Our preliminary data shows that patients who develop AKI are often continued on nephrotoxic medications. Observational, retrospective data from Yale New Haven Hospital, Yale New Haven – St. Raphael campus, and Bridgeport Hospital examined 170,502 admissions that occurred over a three-year period. We identified 29,027 individuals with AKI, a 17% incidence rate that mirrors many prior studies. [22-26] Using time-varying Cox proportional hazards modeling, accounting for age, sex, race, medical versus surgical status, and creatinine, NSAIDs (HR 1.33, 95% CI 1.23-1.44), RAAS agents (HR 1.49, 95% CI 1.43-1.55), and PPIs (HR 1.43, 95% CI 1.39 – 1.48) were associated with the development of AKI. At AKI onset, 4.2% of patients had recently been exposed to an NSAID, 18% to a RAAS agent, and 39% to a PPI. Concerningly, 59%, 66%, and 92% continued to receive their NSAID, ACE/ARB, or PPI respectively, in the 24 hours after AKI development. Taken together, this data suggests that medication-targeted alerts could be beneficial. However, there have been no observational or randomized studies assessing the effect of discontinuing such agents after the development of AKI. In the alerting

framework, randomization to alert status can allow an unbiased assessment of the true effect of medication discontinuation, apart from any confounding factors, on patient outcomes.

Our research group recently conducted a large-scale multicenter randomized controlled trial of electronic alerts for AKI throughout the Yale New Haven Health System from 2018 to 2020. The trial, which enrolled 6,030 patients with AKI, as defined by an increase in creatinine of 0.3mg/dL over 48 hours or 50% over 7 days, randomized patients between usual care and an intervention group whereby providers received a general AKI alert informing them to the presence of AKI and the patient's recent creatinine trends, and provided a link to an AKI-specific order set. Our study showed that, overall, alerting physicians to the presence of AKI did not demonstrate a difference in the rate of our primary outcome of progression of AKI, dialysis, or death, nor were there any differences in process measures accessed (i.e. provider actions) between the two groups, however, there was substantial heterogeneity among the study sites. Stratification of results by hospital revealed that signals of harm exist in the two non-teaching hospitals of our study, where a higher relative risk for our primary outcome was seen in the intervention group. This increased risk was shown to be driven by an increased number of deaths in the alert group, however, process measures still remained unchanged. The alert in this study was limited by the fact that it was purely informational. While an order set was provided, there were no associated patient-specific recommendations or guidance. Given the highly heterogenous nature of AKI, a more personalized approach may be warranted. Further, this study enrolled all patients who developed AKI rather than a targeted subset of patients who may benefit, such as those AKI patients receiving potentially harmful kidney-toxic medications.

In the present proposal, we seek to expand upon our prior study to determine if the use of medication-targeted electronic alerts will modify provider behavior, particularly in regards to nephrotoxic medication use and cessation, in the care of hospitalized patients with AKI and/or reduce the rates of progression to AKI, dialysis, or mortality in hospitalized patients. Given the heterogenous and surprising results of our prior study, it is of utmost importance that the efficacy of such interventions, even those that are seemingly benign, are rigorously evaluated in the setting of a randomized controlled trial.

Therefore, we propose a randomized, controlled trial of a medication-targeted electronic AKI alert system. Using the Kidney Disease: Improving Global Outcomes (KDIGO) creatinine criteria, inpatients at 4 different teaching hospitals of the Yale New Haven Health System that have had at least one dose of a nephrotoxic agent of interest within 24 hours of AKI onset will be randomized to either usual care or a medication-targeted alert that informs the provider of the presence of AKI and the patient's recent exposures to the targeted classes of medications with an option to discontinue. The primary outcome will be a composite of AKI progression, dialysis, or mortality within 14 days of randomization. Secondary outcomes will focus on the rate of cessation of any medication of interest within 24 hours of randomization and various other best practice metrics defined below.

4. **Research Plan:**

Overview:

This study will be a randomized, single blind interventional trial designed to determine the efficacy of a medication-targeted electronic alert versus standard care in reducing nephrotoxic medication administration among hospitalized patients who have developed AKI and have had at least a single dose of a targeted medication within 24 hours prior to AKI onset. Our study flow is the following:

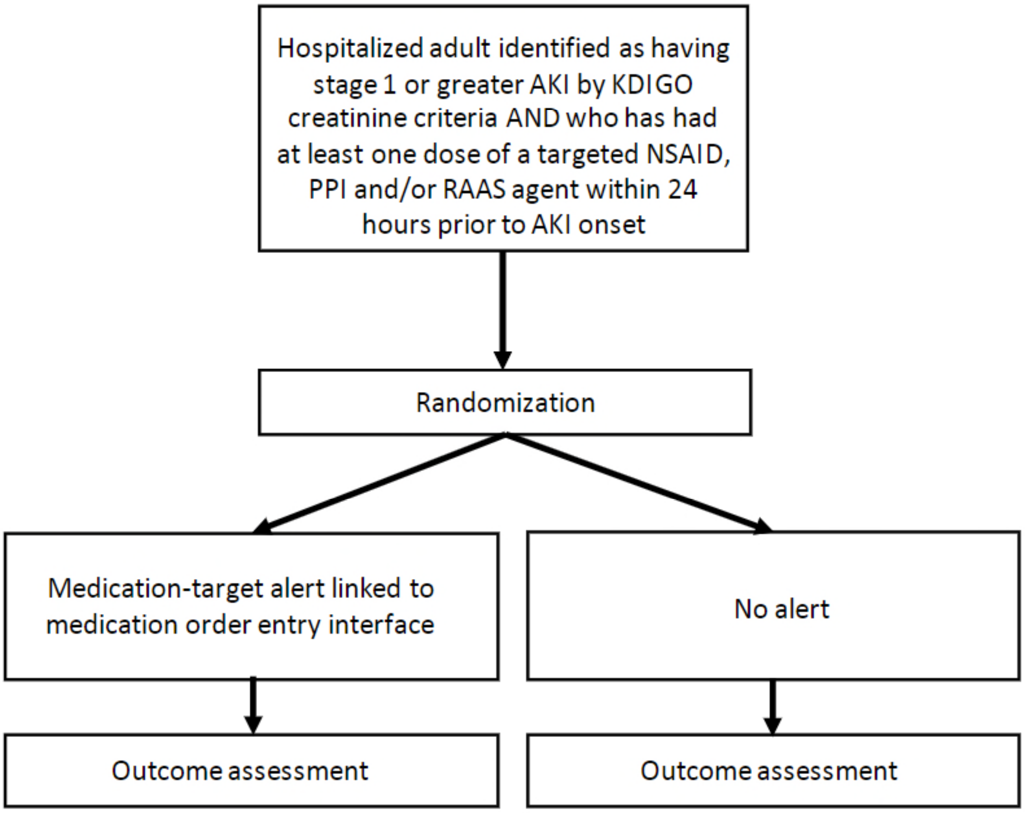

Subject Eligibility and Randomization:

Identification of subjects with AKI will be performed entirely within the Epic medical records system based on inclusion and exclusion criteria outlined below. This will be done using a best-practice alert build developed by the JDAT team at Yale. It works by examining the most recent creatinine value and comparing it to the minimum value in the past 48 hours or the past 7 days. If the current value is 0.3 mg/dl above the 48-hour minimum, or 50% higher than the 7 day minimum, the patient will be defined as having AKI. If, in addition, the patient has an active order for one of our targeted nephrotoxic agents, he or she will be automatically enrolled. Enrolled patients will automatically and immediately be randomized either into the intervention group or the usual care group. Randomization will be done within Epic via a simple randomization method using concealed allocation via a random number rule that is incorporated in the alert. This ensures that, upon meeting criteria, each patient is immediately and randomly assigned to an arm. Logic checks within the alerts ensure that once a patient is assigned to an arm, they remain on that arm for the remainder of their hospital stay.

Study Intervention:

Patients in the intervention (alert) group will have an alert generated within the electronic medical record system. This will consist of a "pop-up" within Epic when a provider accesses the patient's record and opens the order entry screen such that it appears at the most relevant place in a provider's workflow. This alert notifies the physician that the patient has AKI and has recently been given at least one dose of a nephrotoxic medication(s). It also explains that this alert does not fire for all patients and that the patient is part of a randomized trial. A link to our study website is provided. The alert outlines each targeted medication that

the patient is on, and links to the medication entry system. An example of what this alert will look like is as follows:

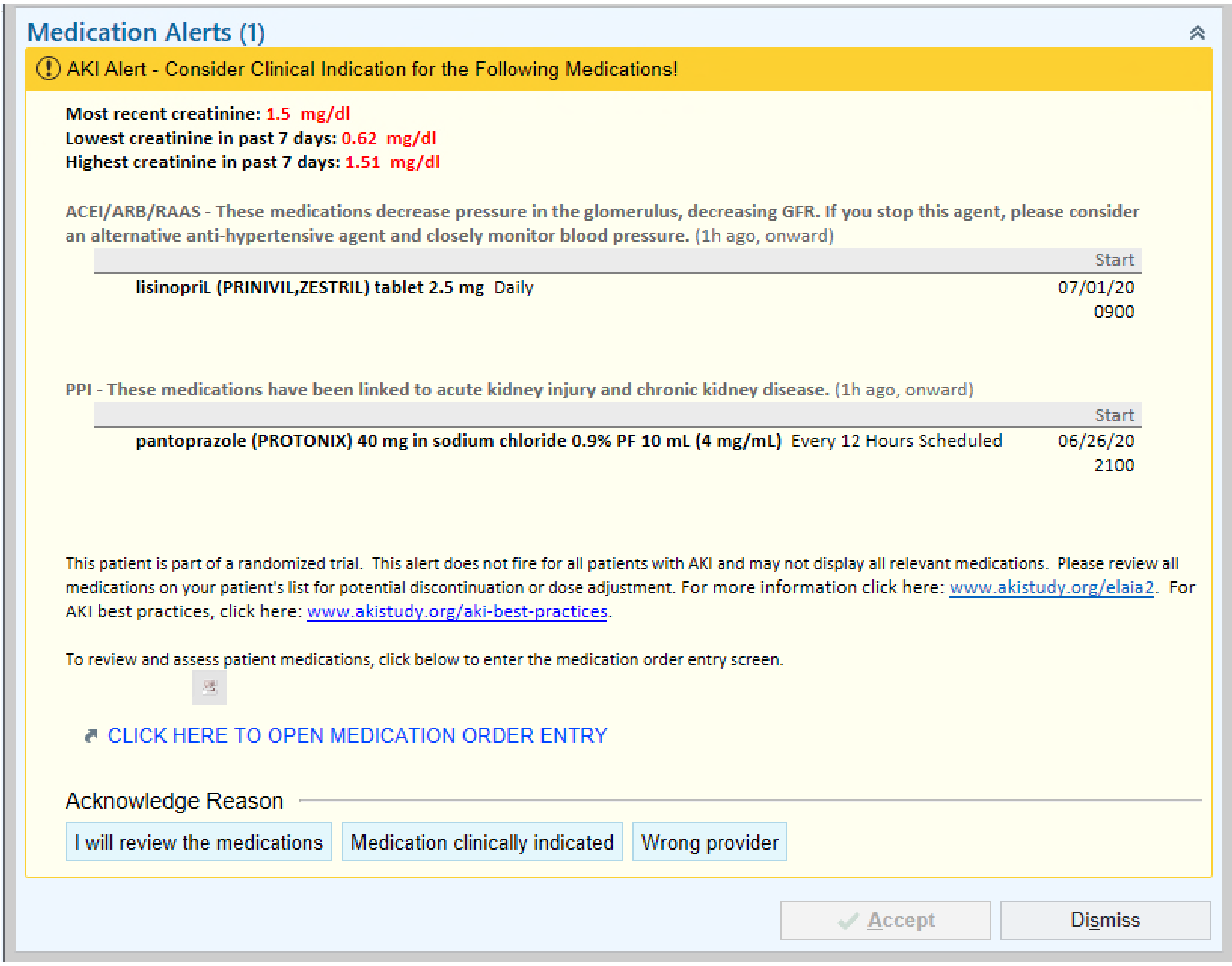

This alert will be received by physicians, physician assistants (PA's), nurse practitioners (NPs), advanced practice registered nurses (APRNs), fellows, and residents who open the order entry screen in the medical record of a patient randomized to the alert arm of the trial, regardless of their relationship with the patient.

The provider will have the option to "dismiss" the alert. However, if dismissed, the alert will continue to pop up for the provider on each subsequent opening of the patient's chart. The alert will only stop firing for any given provider under the following conditions:

- The active nephrotoxin order is discontinued
- The provider acknowledges the alert by clicking "Accept". In this case, the alert will be suppressed for this provider only and for 48 hours.
- The most recent creatinine does not meet AKI criteria
- The patient receives an order for hemodialysis, continuous renal replacement therapy, or peritoneal dialysis
- The patient is transferred to hospice service
- The patient is discharged from the hospital
- The patient expires

Study Outcomes:

Our primary outcome will be a composite of AKI progression, dialysis (hemodialysis, continuous renal replacement therapy, or peritoneal dialysis), or mortality within 14 days of randomization.

Our main secondary outcome will look at the cessation of any of the targeted medications within 24 hours of AKI onset. For individuals with active orders for more than one targeted medication, cessation of any will be adequate to meet this endpoint.

Other secondary study endpoints will look at a variety of patient outcomes as well as best practice metrics, including the following:

- Inpatient mortality
- Inpatient dialysis
- Discharged on dialysis
- Percent who progress to stage 2 AKI
- Percent who progress to stage 3 AKI
- AKI duration
- 30-day readmission rate
- Cost of index hospitalization
- "Best practice" outcomes: Proportion achieved per patient during index hospitalization:
    - Contrast administration
    - Fluid administration
    - Aminoglycoside administration
    - NSAID administration/cessation
    - ACEi administration/cessation
    - Urinalysis order
    - Documentation of AKI
    - Monitoring of creatinine
    - Monitoring of urine output
    - Renal consults

All investigators will be blinded to the treatment assignment until the end of the trial period.

Clinician Outreach:

While the unit of randomization is the patient, clinicians are considered subjects of this research. We will engage in pre-trial and periodic outreach to all clinicians who may be exposed to this study, informing them of the nature of the study, the fact that it is a randomized trial, and that alerts do not fire for all patients with AKI. We will additionally inform them that limited data is being collected regarding provider behavior. However, we will also make it clear that data subject to clinician behavior (continuation or discontinuation of any medication) will NOT be linked to individual clinicians. All such data will only be analyzed in aggregate. This outreach will be performed through short presentations at divisional conferences and meetings and through regular visits to hospital floors to engage directly with providers. A study Information Sheet will also be handed out to clinicians.

Study Timeline:

We plan to activate our medication-targeted alert in a step-wise fashion throughout our 4 study hospitals. Staggered enrollment will allow for proper pre-trial education as well as adequate site visits, study monitoring, quality control and clinician engagement at each site as roll out begins.

5. Genetic Testing N/A ☒
   A. Describe
      i. the types of future research to be conducted using the materials, specifying if immortalization of cell lines, whole exome or genome sequencing, genome wide association studies, or animal studies are planned *Write here*
      ii. the plan for the collection of material or the conditions under which material will be received *Write here*
      iii. the types of information about the donor/individual contributors that will be entered into a database *Write here*
      iv. the methods to uphold confidentiality *Write here*

   B. What are the conditions or procedures for sharing of materials and/or distributing for future research projects? *Write here*
   C. Is widespread sharing of materials planned? *Write here*
   D. When and under what conditions will materials be stripped of all identifiers? *Write here*
   E. Can donor-subjects withdraw their materials at any time, and/or withdraw the identifiers that connect them to their materials? *Write here*
      i. How will requests to withdraw materials be handled (e.g., material no longer identified: that is, anonymized) or material destroyed)? *Write here*
   F. Describe the provisions for protection of participant privacy *Write here*
   G. Describe the methods for the security of storage and sharing of materials *Write here*

6. Subject Population: Provide a detailed description of the types of human subjects who will be recruited into this study.
   This study will enroll any hospitalized adult patient currently admitted to any of the four teaching hospitals within the Yale New Haven Hospital system, who has developed incident AKI, and who has also received a nephrotoxic medication within the 24 hours prior to AKI onset. The following are the study's inclusion and exclusion criteria for enrolling subjects.

   Inclusion Criteria
   1. Adults ≥18 years admitted to a teaching hospital within the Yale New Haven Hospital system (Yale New Haven Hospital, St. Raphael's, Bridgeport Hospital, Greenwich Hospital)
   2. Incident Acute kidney injury as defined by Kidney Disease: Improving Global Outcomes (KDIGO) creatinine criteria:
      - 0.3 mg/dL increase in serum creatinine over 52 hours OR
      - 50% relative increase in serum creatinine over 172 hours
   3. Received at least one dose of a pre-specified NSAID, RAAS, or PPI agent within 24 hours prior to AKI onset

   Exclusion Criteria
   1. Dialysis order prior to AKI onset
   2. Initial creatinine ≥ 4.0mg/dL
   3. Prior admission in which patient was randomized
   4. Admission to hospice service or comfort measures only order

5. ESKD diagnosis code
6. Kidney transplant within six months

7. **Subject classification:** Check off all classifications of subjects that will be specifically recruited for enrollment in the research project. Will subjects who may require additional safeguards or other considerations be enrolled in the study? If so, identify the population of subjects requiring special safeguards and provide a justification for their involvement.

☐Children	☐ Healthy	☐Fetal material, placenta, or dead fetus
☐Non-English Speaking	☐ Prisoners	☐Economically disadvantaged persons
☐Decisionally Impaired	☐ Employees	☐Pregnant women and/or fetuses
☐Yale Students	☐ Females of childbearing potential	

NOTE: Is this research proposal designed to enroll children who are wards of the state as potential subjects?
Yes ☐ No ☒

8. **Inclusion/Exclusion Criteria:** What are the criteria used to determine subject inclusion or exclusion? See above.
9. How will **eligibility** be determined, and by whom? Eligibility will be assessed electronically, without human intervention, within the Epic best practice alert framework as built by JDAT. Those that meet the inclusion criteria and who have no exclusion criteria will be automatically enrolled and randomized.
10. **Risks:** Describe the reasonably foreseeable risks, including risks to subject privacy, discomforts, or inconveniences associated with subjects participating in the research.
The risks to subjects in these trials is minimal, as the studies randomize them to usual care versus an alert that simply synthesizes data that is already present in the medical record. Potential risks include:
- Loss of confidentiality, as we will be collecting patient information and data.
- Overtreatment: If alerts affect physician behavior, then patients randomized to an alert arm may be more likely to undergo certain tests or interventions such as fluid boluses. These interventions fall within the standard-of-care and may benefit patients, but it is also possible that additional interventions may not benefit patients and could incur additional costs.
- Under-treatment: Patients randomized to medication-targeted alerts may have these medications discontinued more often. This should be beneficial, as the medications chosen have been associated with AKI. But it is also possible that, in some situations, discontinuation of medicine might engender risk (for example, discontinuation of a proton-pump inhibitor among patients with a gastrointestinal bleed). The internal DSMB has identified the following risks of discontinuation for each medication class with as associated monitoring plan to ensure that differential discontinuation does not cause harm to patients:

Nephrotoxin	Risk(s) of Discontinuation	Monitoring Plan
NSAID	-Increased use of opioid analgesics<br>-Worse pain	-Morphine-equivalent opioid exposure per patient-day<br>-Use of naloxone and/or naltrexone<br>-Intubation<br>-Nursing-reported pain score
ACE/ARB/Renin	-Hypertension<br>-Pulmonary Edema<br>-Heart Failure	-Blood pressure<br>-Intubation<br>-Chest X-Rays

		-Echocardiogram
PPI	-GI Bleed<br>-Pain<br>-Severe anemia	-Blood Transfusion<br>-Endoscopy Procedures<br>-Nursing-reported pain score

- Inattention to AKI: It is possible that, as providers learn that alerts exist, they become less attentive to the presence of AKI in patients randomized to the usual care group. We limit this risk by including the language "THIS ALERT DOES NOT FIRE FOR ALL PATIENTS WITH AKI" in each alert. Additionally, our educational efforts (which target each hospital division before the trial and biannually after initiation) make explicit the fact that these alerts are being conducted in a randomized framework.

-Alert Fatigue: These studies represent an additional alert to which providers will be exposed and prior research has demonstrated that more frequent alerting may lead to less attention to other alerts. While a potential risk, this is also a major motivation of this line of research, as only via randomized trials can truly effective alerts be discovered. Should no effect be found in these studies, AKI alerts will not be continued at the institutions.

- Risk of increased signals of harm in the alert group. As discussed above, our recent AKI alert trial showed signals of harm of our general AKI alert, as shown by an increased relative risk of the primary outcome, in the two non-teaching hospitals of our study. Because of this, we will no longer be enrolling in the non-teaching hospitals for this study.

11. **Minimizing Risks:** Describe the manner in which the above-mentioned risks will be minimized.
    - There is limited risk to the loss of confidentiality, as only de-identified data is being stored for analysis. All data in this study will be stored on a central server within the Program of Applied Translational Research. The server is only accessible from within the Yale intranet (or via VPN remotely) and additionally requires separate logon username and password.

      Data abstracted from the medical record will be de-identified, with a linking file retained in a separate location that will allow for future linking of de-identified data to protected health information (PHI) for the purpose of potential future studies. Studies that require the use of PHI (for example, linking patient info to national outcomes databases) will require approval of both the manuscript and executive committees and a separate IRB approval. De-identified data will be stored on a secure server, accessed via "dumb" terminals, and all analyses will proceed on that server alone. De-identified data will be transmitted to outside investigators using secure, encrypted channels upon approval of the manuscript committee.

      The study is not causing any novel data to be gathered about any patient; it only gathers data from already-existing electronic health records. No PHI are included in the data being analyzed or published. Study participants therefore face no greater dangers of loss of confidentiality than they already face as patients.

      This data is not subject to any data sharing agreements at this time. As this will be a large and useful data set that may be utilized in future research protocols, we plan to maintain both the de-identified data set and the linking dataset for at least the duration of IRB approval, and will seek permission to maintain those files on a yearly basis from the IRB if the research protocol is renewed.

- To minimize the risk of undertreatment, the internal DSMB has identified outcomes of interest for each of the medication classes to ensure that differential discontinuation is not causing harm and will utilize their authority to suspend an arm of the study should harm be detected. Additionally, medication-targeted alerts will contain language that illustrates patient populations wherein continuation of the medication may be warranted (please refer to Data and Safety Monitoring Plan document).

- To limit the risk of inattention to AKI, we include the following language on the alert screen: "THIS ALERT DOES NOT FIRE FOR ALL PATIENTS WITH AKI". Additionally, our educational efforts (which target each hospital division before the trial and biannually after initiation) make explicit the fact that these alerts are being conducted in a randomized framework.

- Based on the new information obtained from our recent general AKI alert study which showed signals of harm in the non-teaching hospitals, we are taking necessary precautions to maximize patient safety in this trial. This proposed trial does involve a unique and more specific population than that of our prior study, and utilizes a different intervention which offers more individualized recommendations and guidance for clinicians.

  As a preliminary assessment of safety of our drug-targeted alert, we analyzed a subset of data from our initial alert trial, including only patients who meet criteria for the current study (those AKI patients who had at least one dose of one of our targeted nephrotoxic medications within the 24 hours prior to AKI development). The results, both overall and stratified by hospital, are shown below:

Outcome Table

Variables	Control (N=1244)	Alert (N=1250)	p-value
**Primary Outcome**			.
AKI Progression, Dialysis or Death	205 (16.5%)	234 (18.7%)	0.1379
**Secondary Outcomes**			.
AKI Progression	162 (13%)	194 (15.5%)	0.0762
Dialysis	29 (2.3%)	35 (2.8%)	0.4714
Death	73 (5.9%)	67 (5.4%)	0.6142
Discharge to Home	688 (55.3%)	660 (52.8%)	0.1791
Length of Stay, days after randomization	3.4 (1.6,7.7)	4 (1.9,8.2)	0.3585
**Duration of AKI, days**			0.8928
<2 days	926 (74.4%)	930 (74.4%)	.
2 - <7 days	275 (22.1%)	272 (21.8%)	.
7+ days	43 (3.5%)	48 (3.8%)	.
**Max AKI Stage Achieved**			0.8527
Stage 0	2 (0.2%)	2 (0.2%)	.
Stage 1	985 (79.2%)	982 (78.6%)	.
Stage 2	155 (12.5%)	165 (13.2%)	.
Stage 3	102 (8.2%)	101 (8.1%)	.

Outcome Table
YNH

Variables	Control (N=462)	Alert (N=449)	p-value
**Primary Outcome**			.
AKI Progression, Dialysis or Death	92 (19.9%)	109 (24.3%)	0.1124
**Secondary Outcomes**			.
AKI Progression	73 (15.8%)	98 (21.8%)	0.0199
Dialysis	14 (3%)	18 (4%)	0.4225
Death	35 (7.6%)	27 (6%)	0.3492
Discharge to Home	263 (56.9%)	244 (54.3%)	0.4326
Length of Stay, days after randomization	4.4 (2.2,10.1)	4.5 (2.3,10.3)	0.4344
**Duration of AKI, days**			0.5453
<2 days	328 (71%)	319 (71%)	.
2 - <7 days	115 (24.9%)	105 (23.4%)	.
7+ days	19 (4.1%)	25 (5.6%)	.
**Max AKI Stage Achieved**			0.2401
Stage 0	2 (0.4%)	0 (0%)	.
Stage 1	350 (75.8%)	322 (71.7%)	.
Stage 2	68 (14.7%)	78 (17.4%)	.
Stage 3	42 (9.1%)	49 (10.9%)	.

Outcome Table
SRC

Variables	Control (N=282)	Alert (N=293)	p-value
**Primary Outcome**			.
AKI Progression, Dialysis or Death	39 (13.8%)	43 (14.7%)	0.7718
**Secondary Outcomes**			.
AKI Progression	32 (11.3%)	39 (13.3%)	0.4744
Dialysis	4 (1.4%)	9 (3.1%)	0.1825
Death	10 (3.5%)	7 (2.4%)	0.4129
Discharge to Home	167 (59.2%)	169 (57.7%)	0.7079
Length of Stay, days after randomization	3.2 (1.3,7.1)	3.2 (1.3,6.8)	0.9256
**Duration of AKI, days**			0.9270
<2 days	215 (76.2%)	220 (75.1%)	.
2 - <7 days	56 (19.9%)	60 (20.5%)	.
7+ days	11 (3.9%)	13 (4.4%)	.
**Max AKI Stage Achieved**			0.7322
Stage 0	0 (0%)	1 (0.3%)	.
Stage 1	232 (82.3%)	235 (80.2%)	.
Stage 2	27 (9.6%)	30 (10.2%)	.
Stage 3	23 (8.2%)	27 (9.2%)	.

Outcome Table
BH

Variables	Control (N=188)	Alert (N=213)	p-value
**Primary Outcome**			.
AKI Progression, Dialysis or Death	38 (20.2%)	32 (15%)	0.1719
**Secondary Outcomes**			.
AKI Progression	34 (18.1%)	26 (12.2%)	0.0996
Dialysis	6 (3.2%)	4 (1.9%)	0.3999
Death	10 (5.3%)	9 (4.2%)	0.6069
Discharge to Home	109 (58%)	116 (54.5%)	0.4786
Length of Stay, days after randomization	3.7 (1.4,7.3)	4.3 (1.6,8.2)	0.3208
**Duration of AKI, days**			0.8373
<2 days	137 (72.9%)	154 (72.3%)	.
2 - <7 days	43 (22.9%)	52 (24.4%)	.
7+ days	8 (4.3%)	7 (3.3%)	.
**Max AKI Stage Achieved**			0.0304
Stage 0	0 (0%)	1 (0.5%)	.
Stage 1	137 (72.9%)	179 (84%)	.
Stage 2	31 (16.5%)	20 (9.4%)	.
Stage 3	20 (10.6%)	13 (6.1%)	.

Outcome Table
GH

Variables	Control (N=142)	Alert (N=131)	p-value
**Primary Outcome**			.
AKI Progression, Dialysis or Death	17 (12%)	24 (18.3%)	0.1424
**Secondary Outcomes**			.
AKI Progression	9 (6.3%)	15 (11.5%)	0.1361
Dialysis	2 (1.4%)	1 (0.8%)	0.6095
Death	9 (6.3%)	10 (7.6%)	0.6743
Discharge to Home	76 (53.5%)	58 (44.3%)	0.1268
Length of Stay, days after randomization	2.5 (1.3,5.2)	3.2 (1.3,7.9)	0.1617
**Duration of AKI, days**			0.7278
<2 days	113 (79.6%)	101 (77.1%)	.
2 - <7 days	27 (19%)	29 (22.1%)	.
7+ days	2 (1.4%)	1 (0.8%)	.
**Max AKI Stage Achieved**			0.1716
Stage 0	120 (84.5%)	108 (82.4%)	.
Stage 1	14 (9.9%)	20 (15.3%)	.
Stage 2	8 (5.6%)	3 (2.3%)	.

Outcome Table
LMH

Variables	Control (N=124)	Alert (N=125)	p-value
**Primary Outcome**			.
AKI Progression, Dialysis or Death	15 (12.1%)	22 (17.6%)	0.2222
**Secondary Outcomes**			.
AKI Progression	10 (8.1%)	13 (10.4%)	0.5245
Dialysis	3 (2.4%)	3 (2.4%)	0.9921
Death	9 (7.3%)	12 (9.6%)	0.5061
Discharge to Home	54 (43.5%)	56 (44.8%)	0.8424
Length of Stay, days after randomization	4 (2.1,8.3)	3.1 (1.2,7)	0.1311
**Duration of AKI, days**			0.6243
<2 days	93 (75%)	100 (80%)	.
2 - <7 days	29 (23.4%)	23 (18.4%)	.
7+ days	2 (1.6%)	2 (1.6%)	.
**Max AKI Stage Achieved**			0.7904
Stage 0	107 (86.3%)	104 (83.2%)	.
Stage 1	10 (8.1%)	12 (9.6%)	.
Stage 2	7 (5.6%)	9 (7.2%)	.

Outcome Table
WH

Variables	Control (N=46)	Alert (N=39)	p-value
**Primary Outcome**			.
AKI Progression, Dialysis or Death	4 (8.7%)	4 (10.3%)	0.8060
**Secondary Outcomes**			.
AKI Progression	4 (8.7%)	3 (7.7%)	0.8668
Dialysis	0 (0%)	2 (5.1%)	0.1201
Death	19 (41.3%)	17 (43.6%)	0.8317
Discharge to Home	2.3 (1.2,5.8)	3.7 (1.2,5.9)	0.9402
Length of Stay, days after randomization			0.5650
**Duration of AKI, days**	40 (87%)	36 (92.3%)	.
<2 days	5 (10.9%)	3 (7.7%)	.
2 - <7 days	1 (2.2%)	0 (0%)	.
7+ days			0.4111
**Max AKI Stage Achieved**	39 (84.8%)	34 (87.2%)	.
Stage 0	5 (10.9%)	5 (12.8%)	.
Stage 1	2 (4.3%)	0 (0%)	.

As can be seen in the tables above, there is no evidence of harm or increased risk of relative risk of our primary outcome in the intervention group within this population of AKI patients from our prior alert trial. While we understand that this is no guarantee of harm in the current trial, it suggests that an AKI alert may have no negative effects on this subgroup of patients.

As discussed above, we plan to do a staggered enrollment across our study sites and plan to perform an interim analysis at 50% enrollment at the teaching hospitals. Any signal of harm will suspend the study to further enrollment.

12. **Data and Safety Monitoring Plan:** Include an appropriate Data and Safety Monitoring Plan (DSMP) based on the investigator's risk assessment stated below. (Note: the HIC will make the final determination of the risk to subjects.)
    a. What is the investigator's assessment of the overall risk level for subjects participating in this study? This protocol presents minimal risk to all subjects.
    b. If children are involved, what is the investigator's assessment of the overall risk level for the children participating in this study? N/A
    c. Include an appropriate Data and Safety Monitoring Plan. Examples of DSMPs are available here http://your.yale.edu/policies-procedures/forms/420-fr-01-data-and-safety-monitoring-plans-templates for
        i. Minimal risk
        ii. Greater than minimal

The principal investigator is responsible for monitoring the data, assuring protocol compliance, and conducting the safety reviews biannually. During the review process the principal investigator will evaluate whether the study should continue unchanged, require modification/amendment, or close to enrollment. The principal investigator and the Institutional Review Board (IRB) have the authority to stop or suspend the study or require modifications.

Based on new information obtained from our recent general AKI alert study, we are taking necessary precautions to maximize patient safety in this trial. While we still feel that this is a minimal risk trial, we plan to have a Data Safety Monitoring Board review our results at an interim analysesat 50% enrollment across our four teaching hospitals. Any signal of harm will suspend the study to further enrollment.

This protocol presents minimal risks to the subjects and Unanticipated Problems Involving Risks to Subjects or Others (UPIRSOs), including adverse events, are not anticipated. In the unlikely event that such events occur, Reportable Events (which are events that are serious or life-threatening and unanticipated (or anticipated but occurring with a greater frequency than expected) and possibly, probably, or definitely related) or Unanticipated Problems Involving Risks to Subjects or Others that may require a temporary or permanent interruption of study activities will be reported immediately (if possible), followed by a written report within 5 calendar days of the Principal Investigator becoming aware of the event to the IRB (using the appropriate forms from the website) and any appropriate funding and regulatory agencies. The investigator will apprise fellow investigators and study personnel of all UPIRSOs and adverse events that occur during the conduct of this research project through regular study meetings and via email as they are reviewed by the principal investigator. The protocol's research monitor(s), e.g., DSMBs, study sponsors, funding and regulatory agencies, and regulatory and decision-making bodies will be informed of all adverse events within 5 days of the event becoming known to the principal investigator.

d. For multi-site studies for <u>which the Yale PI serves as the lead investigator</u>:
   i. How will adverse events and unanticipated problems involving risks to subjects or others be reported, reviewed and managed? All adverse events and unanticipated problems will be handled based on the protocols in place by each institution's IRB.
   ii. What provisions are in place for management of interim results? Interim results will be managed by study personnel at Yale.
   iii. What will the multi-site process be for protocol modifications? Any protocol modifications will be submitted to each institution's IRB for the appropriate approvals.

13. **Statistical Considerations:** Describe the statistical analyses that support the study design.
A group sequential design with Hwang-Shih-DeCani spending functions with gamma = -4 for the efficacy boundary and gamma = -2 for the futility boundary with two interim analyses will be utilized and a two-sided Cochran-Mantel-Haenszel (CMH) method will be used for the sample size calculation to account for the four hospitals. Our preliminary data suggests that 18.2% of patients would experience the primary outcome of progression of AKI, dialysis, or mortality. We determined that a relative 20% reduction to 14.6% would be clinically significant. A total sample size of 4600 patients (i.e., 2301 per group) achieves at least 90% power to detect this degree of change. The alpha significance level of the test was set at 0.05. We will increase this

number by 10% to account for potential contamination of the effect across study arms, leading to a final sample size of 5060 individuals with a breakdown as follows based on relative numbers of beds in each hospital:
Yale New Haven Hospital: 2447
St. Raphael's Campus: 1214
Bridgeport Hospital: 910
Greenwich Hospital: 489

We will compare the rates of the primary outcomes between the intervention arm and the control arm using the chi-square test with Cochran-Mantel-Haenszel correction for the 4 study strata based on the hospital.. We will also perform exploratory analyses examining patients within the three medication categories to assess whether discontinuation rates differ by medication and, importantly, what clinical impact is apparent from alerting for those particular agents. Finally, should alerting show a clinical benefit, we will perform a mediation analysis to determine what fraction of that benefit (if any) can be attributed to medication cessation.

In addition, a retrospective examination will be performed to assess improvement in the control group before / after intervention as a measurement of contamination. We expect that providers who receive AKI alerts on some patients may be more likely to look for AKI in other patients, potentially diluting the effect of the intervention – this pre/post intervention analysis will help in assessing that level of contamination.

Because we are enrolling patients across four sites, we will do an exploratory analysis to determine the efficacy of alerts independently at each hospital.

We have specified several subgroups in whom the benefit of an AKI alert may differ from the general population. These groups will be analyzed as secondary and exploratory analyses. They include:

- Patients with AKI exposed to each of the 3 nephrotoxin categories (effects analyzed separately)
- Surgical patients (defined by admission to a surgical team)
- Subjects with baseline creatinine <1.0mg/dl
- Subjects with baseline creatinine <0.5 mg/dl
- Females (due to lower rate of increase in creatinine after AKI)
- African Americans (due to higher rate of increase in creatinine after AKI)
- Elderly subjects (age > 65, age > 70, and age > 75)
- Subjects in an ICU at the time of the alert
- Subjects who enter the study based on a 50% increase in creatinine vs. a 0.3mg/dl increase in creatinine vs. both

Finally, as the academic year progresses, new clinicians (such as residents and interns) may become more facile in their ability to recognize AKI, which may attenuate the effect of this intervention. We will model this in exploratory analyses using an intervention-by-time of year interaction term in models of the primary outcome.

Interim Analysis:

As discussed above, we plan an interim analysis, and guidelines will be given to the DSMB for stopping the trial will be for efficacy, harm, and futility. Our interim analyses will be conducted as follows:

This interim analysis will be performed at 50% enrollment at the four teaching hospitals and the trial will stop for either efficacy or futility if the p-value is <= .0042 or >= .8952. The p-value threshold for the final analysis of the primary outcome is set at .0498.

SECTION II: RESEARCH INVOLVING DRUGS, BIOLOGICS, RADIOTRACERS, PLACEBOS AND DEVICES

If this section (or one of its parts, A or B) is not applicable, check off N/A and delete the rest of the section.

A. RADIOTRACERS ☒N/A

B. DRUGS/BIOLOGICS ☒N/A

B. DEVICES ☒N/A

SECTION III: RECRUITMENT/CONSENT AND ASSENT PROCEDURES

1. **Targeted Enrollment: Give the number of subjects:**
   a. Targeted for enrollment at Yale for this protocol: 1915 patients and approximately 2500 clinicians
   b. If this is a multi-site study, give the total number of subjects targeted across all sites: 4592 patients and approximately 3500 clinicians

2. **Indicate recruitment methods below.** Attach copies of any recruitment materials that will be used.

☐ Flyers
☐ Posters
☐ Letter
☒ Medical record review*
☐ Departmental/Center newsletters
☐ YCCI Recruitment database
☐ Other:
☐ Internet/web postings
☐ Mass email solicitation
☐ Departmental/Center website
☐ Departmental/Center research boards
☐ Web-based clinical trial registries
☐ Social Media (Twitter/Facebook):
☐ Radio
☐ Telephone
☐ Television
☐ Newspaper
☐ Clinicaltrails.gov

* **Requests for medical records should be made through JDAT as described at** http://medicine.yale.edu/ycci/oncore/availableservices/datarequests/datarequests.aspx

3. **Recruitment Procedures:**

a. Describe how potential subjects will be identified. Subjects (patients) will be identified using an electronic algorithm that interfaces with Epic databases.

b. Describe how potential subjects are contacted. There will be no formal contact between patient study subjects and study personnel. We will be requesting a waiver of informed consent for patients. To educate providers of the study, we will hold pre-trial informational sessions at divisional conferences and meetings in which we will explain the purpose of the study and what to expect. We will provide an informational sheet with all relevant information and our contact information. We will also make periodic visits to the hospital floors to reeducate and answer any questions.

c. Who is recruiting potential subjects? There will be no formal recruitment activities. Rather, patient subjects will be identified and enrolled into the study via an electronic algorithm embedded in Epic. All providers will be potentially exposed to the alerts and the P.I. and research coordinator of the study will educate providers as explained above.

4. **Assessment of Current Health Provider Relationship for HIPAA Consideration:**
   Does the Investigator or any member of the research team have a direct existing clinical relationship with any potential subject?
   ☐Yes, all subjects

☐Yes, some of the subjects
☒No

If yes, describe the nature of this relationship. N/A

5. **Request for waiver of HIPAA authorization:** (When requesting a waiver of HIPAA Authorization for either the entire study, or for recruitment purposes only. Note: if you are collecting PHI as part of a phone or email screen, you must request a HIPAA waiver for recruitment purposes.)
   Choose one:
   ☒ For entire study
   ☐ For recruitment/screening purposes only
   ☐ For inclusion of non-English speaking subject if short form is being used and there is no translated HIPAA research authorization form available on the University's HIPAA website at hipaa.yale.edu.

   i. Describe why it would be impracticable to obtain the subject's authorization for use/disclosure of this data: Due to the nature of the study in that we are assessing the influence of an alert system on provider behaviors surrounding AKI, it is not feasible to consent subjects as it would severely contaminate the exposure of interest. Patients randomized to the control arm would need to be told not to inform their provider of their participation in the trial, which would severely compromise the therapeutic relationship. The only way to avoid this would be to obtain informed consent from every patient on admission to the hospital, but given that the incidence of AKI is approximately 15% of admissions, this would be impractical and would be unnecessary for the vast majority of patients. Furthermore, it would expose the roughly 85% of patients who would never be deemed eligible for the trial to the risks of loss of confidentiality present in all research with human subjects. Given that the research proposed presents no more than minimal risk to the subjects, and that no procedures are being performed that require consent outside of the research paradigm, we request a HIPAA waiver from the Institutional Review Board.

   ii. If requesting a waiver of **signed** authorization, describe why it would be impracticable to obtain the subject's signed authorization for use/disclosure of this data: *Write here*

The investigator assures that the protected health information for which a Waiver of Authorization has been requested will not be reused or disclosed to any person or entity other than those listed in this application, except as required by law, for authorized oversight of this research study, or as specifically approved for use in another study by an IRB.

Researchers are reminded that unauthorized disclosures of PHI to individuals outside of the Yale HIPAA-Covered entity must be accounted for in the "accounting for disclosures log", by subject name, purpose, date, recipients, and a description of information provided. Logs are to be forwarded to the Deputy HIPAA Privacy Officer.

6. **Process of Consent/Assent:** Describe the setting and conditions under which consent/assent will be obtained, including parental permission or surrogate permission and the steps taken to ensure subjects' independent decision-making.
   Applicable to clinician subjects – we will perform outreach through short presentations at divisional conferences and meetings and through regular visits to hospital floors to engage directly with providers. A study Information Sheet will also be handed out to clinicians.
7. **Evaluation of Subject(s) Capacity to Provide Informed Consent/Assent:** Indicate how the personnel obtaining consent will assess the potential subject's ability and capacity to consent to the research being proposed.

N/A

8. **Non-English Speaking Subjects:** Explain provisions in place to ensure comprehension for research involving non-English speaking subjects. If enrollment of these subjects is anticipated, translated copies of all consent materials must be submitted for approval prior to use.
N/A

As a limited alternative to the above requirement, will you use the short form* for consenting process if you unexpectedly encounter a non-English speaking individual interested in study participation and the translation of the long form is not possible prior to intended enrollment? YES ☐ NO ☒

> **Note*** If more than 2 study participants are enrolled using a short form translated into the same language, then the full consent form should be translated into that language for use the next time a subject speaking that language is to be enrolled.
>
> Several translated short form templates are available on the HRPP website (yale.edu/hrpp) and translated HIPAA Research Authorization Forms are available on the HIPAA website (hipaa.yale.edu). If the translation of the short form is not available on our website, then the translated short form needs to be submitted to the IRB office for approval via modification prior to enrolling the subject. ***Please review the guidance and presentation on use of the short form available on the HRPP website.***
>
> **If using a short form without a translated HIPAA Research Authorization Form, please request a HIPAA waiver in the section above.**

9. **Consent Waiver: In certain circumstances, the HIC may grant a waiver of signed consent, or a full waiver of consent, depending on the study.** If you will request either a waiver of consent, or a waiver of signed consent for this study, complete the appropriate section below.

☐**Not Requesting any consent waivers**

☒**Requesting a waiver of signed consent:**

☐ **Recruitment/Screening only** *(if for recruitment, the questions in the box below will apply to recruitment activities only)*

☒ **Entire Study (**Note that an information sheet may be required.) For clinician subjects.

> **For a waiver of signed consent, address the following:**
> - Would the signed consent form be the only record linking the subject and the research? **YES** ☒ **NO** ☐
> - Does a breach of confidentiality constitute the principal risk to subjects? **YES** ☒ **NO** ☐
>
> **OR**
> - Does the research pose greater than minimal risk? **YES** ☐ **NO**☐
> - Does the research include any activities that would require signed consent in a non-research context? **YES** ☐ **NO** ☐

☒ **Requesting a waiver of consent:**

☐ **Recruitment/Screening only** *(if for recruitment, the questions in the box below will apply to recruitment activities only)*

☒ **Entire Study** - for patient subjects

For a full waiver of consent, please address all of the following:

- Does the research pose greater than minimal risk to subjects?
  ☐ Yes *If you answered yes, stop. A waiver cannot be granted.*
  ☒ No
- Will the waiver adversely affect subjects' rights and welfare? YES ☐ NO☒
- Why would the research be impracticable to conduct without the waiver? Due to the nature of the study, it is not feasible to consent subjects, as it would severely contaminate the exposure of interest. Patients randomized to the control arm would need to be told not to inform their provider of their participation in the trial, which would severely compromise the therapeutic relationship. The only way to avoid this would be to obtain informed consent from every patient on admission to the hospital, but given that the incidence of AKI is approximately 15% of admissions, this would be impractical and would be unnecessary for the vast majority of patients. Given that the research proposed presents no more than minimal risk to the subjects, and that no procedures are being performed that require consent outside of the research paradigm, we will request a waiver of consent from the Institutional Review Board.
- Where appropriate, how will pertinent information be returned to, or shared with subjects at a later date? The results of this study will not be pertinent to the subjects, as the effect of the alert is expected to be transient and should have no impact on their future quality of life beyond that measured by the outcome of the study itself. Moreover, post-hoc disclosure of the nature of the study to the subjects may generate undue stress and concern on the part of the subject as to the quality of their overall clinical care. As most patients are not familiar with AKI, informing them to the presence of the disease without offering a tangible benefit may further increase anxiety surrounding their overall health. We will update all clinicians of the results of this trial after trial completion. We are not collecting data that would tie a specific clinician to an outcome. For example, while we would inform clinicians that the alerts decreased the use of certain drugs, we will not be able to tell them what their specific rate of drug continuation or discontinuation would be.

SECTION IV: PROTECTION OF RESEARCH SUBJECTS

Confidentiality & Security of Data:

1. What protected health information (medical information along with the HIPAA identifiers) about subjects will be collected and used for the research? Patient Name, SSN, zip code, DOB, Admission/discharge dates, medical record number, laboratory values, provider notes, medical procedures, medical history, medication lists, imaging results.

2. How will the research data be collected, recorded and stored? All data, both patient and provider, will be collected electronically by an automated algorithm that interfaces with the EPIC user database system, developed by JDAT. Data will be skimmed from the EHR, processed electronically, encrypted, deidentified, and transferred to a secure server for storage and later analysis. As discussed above, the primary dataset will contain no PHI. We will maintain a separate "linking file" that contains all PHI and will be stored on a separate server to limit the risk of accidental disclosure. The linking file is being maintained for potential future linking to national databases of death and dialysis.

3. How will the digital data be stored? ☐CD ☐DVD ☐Flash Drive ☐Portable Hard Drive ☒Secured Server ☐Laptop Computer ☐Desktop Computer ☐Other

4. What methods and procedures will be used to safeguard the confidentiality and security of the identifiable study data and the storage media indicated above during and after the subject's participation in the study? All data will be stored on an encrypted, HIPAA-Compliant server with 2-factor authentication. Data will not be stored on personal computing devices of any kind. The server will only be accessible from within the Yale firewall or via VPN. No portable devices will be used to store study data at any time.

All portable devices must contain encryption software, per University Policy 5100. If there is a technical reason a device cannot be encrypted please submit an exception request to the Information Security, Policy and Compliance Office by clicking on url http://its.yale.edu/egrc or email it.compliance@yale.edu

5. What will be done with the data when the research is completed? Are there plans to destroy the identifiable data? If yes, describe how, by whom and when identifiers will be destroyed. If no, describe how the data and/or identifiers will be secured. PHI will be deleted by study personnel within one year of closing of the study.

6. If appropriate, has a Certificate of Confidentiality been obtained? A Certificate of Confidentiality automatically covers this NIH-funded research.

SECTION V: POTENTIAL BENEFITS

Potential Benefits: Identify any benefits that may be reasonably expected to result from the research, either to the subject(s) or to society at large. (Payment of subjects is not considered a benefit in this context of the risk benefit assessment.)
Patient-Subjects in this study may benefit from their provider being given information about the presence of acute kidney injury concurrent with the usage of nephrotoxic agents. This benefit may be derived from closer monitoring of physiologic status, cessation of the nephrotoxic agents, diagnostic interventions, or other factors. Regardless of the outcome for participants, the results of these studies may lead to significant societal benefit, as positive results would lead to broader adoption of an effective alerting system that would lead to improved treatment for AKI patients, and a negative study would lead to less enthusiastic adoption of ineffective alerting that would otherwise contribute to alert fatigue.

SECTION VI: RESEARCH ALTERNATIVES AND ECONOMIC CONSIDERATIONS

1. **Alternatives:** What other alternatives are available to the study subjects outside of the research?
   Usual hospital care
2. **Payments for Participation (Economic Considerations):** Describe any payments that will be made to subjects, the amount and schedule of payments, and the conditions for receiving this compensation.
   N/A
3. **Costs for Participation (Economic Considerations):** Clearly describe the subject's costs associated with participation in the research, and the interventions or procedures of the study that will be provided at no cost to subjects.
   N/A
4. **In Case of Injury:** This section is required for any research involving more than minimal risk, and for minimal risk research that presents the potential for physical harm (e.g., research involving blood draws).
   N/A

a. Will medical treatment be available if research-related injury occurs? *Write here*

b. Where and from whom may treatment be obtained? *Write here*
c. Are there any limits to the treatment being provided? *Write here*
d. Who will pay for this treatment? *Write here*
e. How will the medical treatment be accessed by subjects? *Write here*

IMPORTANT REMINDERS

Will this study have a billable service? **Yes** ☐ **No** ☒

A billable service is defined as any service rendered to a study subject that, if he/she was not on a study, would normally generate a bill from either Yale-New Haven Hospital or Yale Medical Group to the patient or the patient's insurer. The service may or may not be performed by the research staff on your study, but may be provided by professionals within either Yale-New Haven Hospital or Yale Medical Group (examples include x-rays, MRIs, CT scans, specimens sent to central labs, or specimens sent to pathology). Notes: 1. There is no distinction made whether the service is paid for by the subject or their insurance (Standard of Care) or by the study's funding mechanism (Research Sponsored). 2. This generally includes new services or orders placed in EPIC for research subjects.

If answered, "yes", this study will need to be set up in OnCore, Yale's clinical research management system, for Epic to appropriately route research related charges. Please contact oncore.support@yale.edu

Are there any procedures involved in this protocol that will be performed at YNHH or one of its affiliated entities?
Yes ☐ **No** ☒

If Yes, please answer questions a through c and note instructions below.

a. Does your YNHH privilege delineation currently include the **specific procedure** that you will perform? **Yes** ☐ **No** ☐

b. Will you be using any new equipment or equipment that you have not used in the past for this procedure? **Yes** ☐ **No** ☐

c. Will a novel approach using existing equipment be applied? **Yes** ☐ **No** ☐

If you answered "no" to question 4a, or "yes" to question 4b or c, please contact the YNHH Department of Physician Services (688-2615) for prior approval before commencing with your research protocol.

IMPORTANT REMINDER ABOUT RESEARCH AT YNHH

Please note that if this protocol includes Yale-New Haven Hospital patients, including patients at the HRU, the Principal Investigator and any co-investigators who are physicians or mid-level practitioners (includes PAs, APRNs, psychologists and speech pathologists) who may have direct patient contact with patients on YNHH premises must have medical staff appointment and appropriate clinical privileges at YNHH. If you are uncertain whether the study personnel meet the criteria, please telephone the Physician Services Department at 203-688-2615. **By submitting this protocol as a PI, you attest that you and any co-investigator who may have patient contact has a medical staff appointment and appropriate clinical privileges at YNHH.**

References:

1. Uchino S, Bellomo R, Goldsmith D, Bates S, Ronco C. An assessment of the RIFLE criteria for acute renal failure in hospitalized patients. Critical care medicine. 2006;34(7):1913-1917.
2. Chertow GM, Soroko SH, Paganini EP, et al. Mortality after acute renal failure: models for prognostic stratification and risk adjustment. Kidney Int. 2006;70(6):1120-1126.
3. Vuckovic S, Vujovic KS, Medic B, Srebro D, Mostic D. Prevention of renal complications induced by non-steroidal anti-inflammatory drugs. Curr Med Chem. 2016.
4. Elia C, Graupera I, Barreto R, et al. Severe acute kidney injury associated with non-steroidal anti-inflammatory drugs in cirrhosis: A case-control study. J Hepatol. 2015;63(3):593-600.
5. Pai AB. Keeping kidneys safe: the pharmacist's role in NSAID avoidance in high-risk patients. J Am Pharm Assoc (2003). 2015;55(1):e15-23; quiz e24-15.
6. Faught LN, Greff MJ, Rieder MJ, Koren G. Drug-induced acute kidney injury in children. Br J Clin Pharmacol. 2015;80(4):901-909.
7. Perazella MA, Luciano RL. Review of select causes of drug-induced AKI. Expert Rev Clin Pharmacol. 2015;8(4):367-371.
8. Murray MD, Brater DC. Renal toxicity of the nonsteroidal anti-inflammatory drugs. Annu Rev Pharmacol Toxicol. 1993;33:435-465.
9. Kidney Disease: Improving Global Outcomes (KDIGO) Acute Kidney Injury Work Group. KDIGO Clinical Practice Guideline for Acute Kidney Injury. Kidney International. 2012;2(Suppl.):1-138.
10. Coca SG, Garg AX, Swaminathan M, et al. Preoperative angiotensin-converting enzyme inhibitors and angiotensin receptor blocker use and acute kidney injury in patients undergoing cardiac surgery. Nephrology Dialysis Transplantation. 2013;28(11):2787-2799.
11. Perazella MA, Coca SG. Three feasible strategies to minimize kidney injury in'incipient AKI'. Nature Reviews Nephrology. 2013;9(8):484-490.
12. Muriithi AK, Leung N, Valeri AM, et al. Biopsy-proven acute interstitial nephritis, 1993-2011: a case series. American Journal of Kidney Diseases. 2014;64(4):558-566.
13. Muriithi AK, Leung N, Valeri AM, et al. Clinical characteristics, causes and outcomes of acute interstitial nephritis in the elderly. Kidney Int. 2015;87(2):458-464.
14. Antoniou T, Macdonald EM, Hollands S, et al. Proton pump inhibitors and the risk of acute kidney injury in older patients: a population-based cohort study. CMAJ Open. 2015;3(2):E166-171.
15. Lazarus B, Chen Y, Wilson FP, et al. Proton Pump Inhibitor Use and the Risk of Chronic Kidney Disease. JAMA Intern Med. 2016;176(2):238-246.
16. Xie Y, Bowe B, Li T, Xian H, Balasubramanian S, Al-Aly Z. Proton Pump Inhibitors and Risk of Incident CKD and Progression to ESRD. J Am Soc Nephrol. 2016.
17. Tawadrous D, Shariff SZ, Haynes RB, Iansavichus AV, Jain AK, Garg AX. Use of clinical decision support systems for kidney-related drug prescribing: a systematic review. Am J Kidney Dis. 2011;58(6):903-914.
18. Licurse A, Kim MC, Dziura J, et al. Renal ultrasonography in the evaluation of acute kidney injury: Developing a risk stratification framework. Archives of Internal Medicine. 2010;170(21):1900-1907.
19. Kwon SH, Noh H, Jeon JS, Kim Y, Han DC. An assessment of AKIN criteria for hospital-acquired acute kidney injury: a prospective observational cohort study. Nephron Clinical Practice. 2010;116(3):c217-c223.
20. Weisbord SD, Mor MK, Resnick AL, et al. Prevention, incidence, and outcomes of contrast-induced acute kidney injury. Archives of Internal Medicine. 2008;168(12):1325-1332.
21. Finlay S, Bray B, Lewington A, et al. Identification of risk factors associated with acute kidney injury in patients admitted to acute medical units. Clinical Medicine. 2013;13(3):233-238.
22. Wilson FP, Yang W, Feldman HI. Predictors of Death and Dialysis in Severe AKI: The UPHS-AKI Cohort. Clinical journal of the American Society of Nephrology : CJASN. 2012.

23. Wilson FP, Shashaty M, Testani J, et al. Automated, electronic alerts for acute kidney injury: a single-blind, parallel-group, randomised controlled trial. The Lancet. 2015;385(9981):1966-1974.
24. Ricci Z, Cruz D, Ronco C. The RIFLE criteria and mortality in acute kidney injury: A systematic review. Kidney international. 2008;73(5):538-546.
25. Li S, Krawczeski CD, Zappitelli M, et al. Incidence, risk factors, and outcomes of acute kidney injury after pediatric cardiac surgery: a prospective multicenter study. Critical care medicine. 2011;39(6):1493-1499.
26. Chertow GM, Burdick E, Honour M, Bonventre JV, Bates DW. Acute kidney injury, mortality, length of stay, and costs in hospitalized patients. Journal of the American Society of Nephrology : JASN. 2005;16(11):3365-3370.
27. Wilson FP, Reese PP, Shashaty MG, et al. A trial of in-hospital, electronic alerts for Acute Kidney Injury: Design and rationale. Clinical trials. 2014.
28. O'Brien PCF, T.R. A Multiple Testing Procedure for Clinical Trials. Biometrics. 1979;35(3):549-556.
29. Wilson FP, Reese PP, Shashaty MG, et al. A trial of in-hospital, electronic alerts for acute kidney injury: design and rationale. Clin. Trials. 2014;11(5):521-529.
