# Supplementary material for: A randomized clinical trial assessing the effect of automated medication-targeted alerts on acute kidney injury outcomes
Source: Nat Commun. 2023 May 17;14:2826. doi: 10.1038/s41467-023-38532-3 (PMC10192367; doi:10.1038/s41467-023-38532-3)
Supplement: Supplementary file 3 — Reporting Summary [file 41467_2023_38532_MOESM3_ESM.pdf]

nature portfolio

Corresponding author(s): F. Perry Wilson, MD MSCE

Last updated by author(s): Apr 24, 2023

Reporting Summary

Nature Portfolio wishes to improve the reproducibility of the work that we publish. This form provides structure for consistency and transparency in reporting. For further information on Nature Portfolio policies, see our Editorial Policies and the Editorial Policy Checklist.

Statistics

For all statistical analyses, confirm that the following items are present in the figure legend, table legend, main text, or Methods section.

n/a	Confirmed	
☐	☒	The exact sample size (*n*) for each experimental group/condition, given as a discrete number and unit of measurement
☐	☒	A statement on whether measurements were taken from distinct samples or whether the same sample was measured repeatedly
☐	☒	The statistical test(s) used AND whether they are one- or two-sided<br>*Only common tests should be described solely by name; describe more complex techniques in the Methods section.*
☐	☒	A description of all covariates tested
☐	☒	A description of any assumptions or corrections, such as tests of normality and adjustment for multiple comparisons
☐	☒	A full description of the statistical parameters including central tendency (e.g. means) or other basic estimates (e.g. regression coefficient) AND variation (e.g. standard deviation) or associated estimates of uncertainty (e.g. confidence intervals)
☐	☒	For null hypothesis testing, the test statistic (e.g. *F*, *t*, *r*) with confidence intervals, effect sizes, degrees of freedom and *P* value noted<br>*Give P values as exact values whenever suitable.*
☒	☐	For Bayesian analysis, information on the choice of priors and Markov chain Monte Carlo settings
☒	☐	For hierarchical and complex designs, identification of the appropriate level for tests and full reporting of outcomes
☐	☒	Estimates of effect sizes (e.g. Cohen's *d*, Pearson's *r*), indicating how they were calculated

Our web collection on statistics for biologists contains articles on many of the points above.

Software and code

Policy information about availability of computer code

Data collection	All data were electronically collected through SQL queries of the Epic Electronic Health Record “Clarity” data platform, reflect clinical care and were recorded in a secure, HIPAA compliant server.
Data analysis	All statistical tests were performed in Stata version 15 (College Station, TX), SAS v9.4 (Cary, NC), and R v4.0.1 (Boston, MA). AKI detection algorithm.

For manuscripts utilizing custom algorithms or software that are central to the research but not yet described in published literature, software must be made available to editors and reviewers. We strongly encourage code deposition in a community repository (e.g. GitHub). See the Nature Portfolio guidelines for submitting code & software for further information.

Data

Policy information about availability of data

All manuscripts must include a data availability statement. This statement should provide the following information, where applicable:

- Accession codes, unique identifiers, or web links for publicly available datasets
- A description of any restrictions on data availability
- For clinical datasets or third party data, please ensure that the statement adheres to our policy

The data generated in this study have been deposited in the DataDryad database under accession code https://doi.org/10.5061/dryad.kh189327p. This data can be

accessed and used for scientific research without permission from the authors. The DSMB has approved the release of this deidentified dataset. Source data and statistical analysis plan are provided with this paper.

Human research participants

Policy information about studies involving human research participants and Sex and Gender in Research.

Reporting on sex and gender	We only had access to self-reported sex (not gender) data. We recognize that, when asked, individuals may report their gender, instead of sex, however we are unable to disaggregate this data in the analysis. Therefore, we describe the data as "sex" and use the terms "male" and "female" throughout the manuscript.
Population characteristics	Population characteristics are fully described in Table 1 of the manuscript.
Recruitment	Participants were automatically enrolled under an authorized waiver of informed consent when the electronic AKI detection algorithm first determined they met all eligibility criteria. Given that there was no consent for this study, we feel there is little possibility for self-section bias. As it was a randomized trial, there is little possibility of other biases. Contamination has been addressed in the manuscript.
Ethics oversight	The protocol was carried out according to the principles in the Declaration of Helsinki and approved by the Yale Human Investigations Committee (HIC #2000025786) which approved a waiver of informed consent. A reliance agreement was obtained from the Bridgeport Hospital and Greenwich Hospital IRBs as well.

Note that full information on the approval of the study protocol must also be provided in the manuscript.

Field-specific reporting

Please select the one below that is the best fit for your research. If you are not sure, read the appropriate sections before making your selection.

☒ Life sciences ☐ Behavioural & social sciences ☐ Ecological, evolutionary & environmental sciences

For a reference copy of the document with all sections, see nature.com/documents/nr-reporting-summary-flat.pdf

Life sciences study design

All studies must disclose on these points even when the disclosure is negative.

Sample size	Based on data from our prior trial, we expected the primary outcome to occur in 18.2% of control patients and considered a relative reduction of 20% to 14.6% would be clinically significant. The target sample size of 5,060 individuals provided 90% power to detect a difference at least this large, accounting for two interim analyses and potential 10% contamination of intervention across study arms (as providers exposed to alerts might learn to adjust their care for patients in the usual care arm).
Data exclusions	There were no data exclusions in this study
Replication	This is a large randomized trial. These results can be replicated by another large randomized trial.
Randomization	Allocation was in a 1:1 ratio, achieved via simple randomization within the electronic health record.
Blinding	Participants and providers were necessarily not masked to the intervention, but investigators were.

Reporting for specific materials, systems and methods

We require information from authors about some types of materials, experimental systems and methods used in many studies. Here, indicate whether each material, system or method listed is relevant to your study. If you are not sure if a list item applies to your research, read the appropriate section before selecting a response.

Materials & experimental systems

n/a	Involved in the study	
☒	☐	Antibodies
☒	☐	Eukaryotic cell lines
☒	☐	Palaeontology and archaeology
☒	☐	Animals and other organisms
☐	☒	Clinical data
☒	☐	Dual use research of concern

Methods

n/a	Involved in the study	
☒	☐	ChIP-seq
☒	☐	Flow cytometry
☒	☐	MRI-based neuroimaging

Clinical data

Policy information about clinical studies

All manuscripts should comply with the ICMJE guidelines for publication of clinical research and a completed CONSORT checklist must be included with all submissions.

Clinical trial registration	NCT02771977
Study protocol	The study protocol appears as a supplement to the manuscript
Data collection	Enrollment was automated, with inclusion and exclusion criteria built into the alert system itself and occurred between 08/16/2020 and 11/29/2021. Eligible patients were adults >= 18 years old who were admitted to one of four participating study hospitals (Supplemental Table 2), had AKI as defined by the Kidney Disease: Improving Global Outcomes (KDIGO) serum creatinine criteria (at least 50% increase in creatinine within 7 days or a 0.3mg/dL increase within 48 hours), and had an active order for one or more of the three MOIs.9 Patients were excluded if their initial creatinine after admission was greater than or equal to 4.0 mg/dL or if they had received dialysis within the year prior to meeting the AKI definition (as these may be patients with end-stage kidney disease cared for outside of our health system), if they had been admitted to a hospice service or had an active "comfort measures only" order, if they had a diagnosis code consistent with end-stage kidney disease, or if they had a kidney transplant within the 6 months prior to randomization (as transplant recipients at our institution receive specialist nephrology care during their inpatient stay, regardless of AKI). Patients who had an admission date prior to the "go-live" date of the alert, who triggered randomization after a discharge order had been entered or had already been randomized during a previous hospitalization were also excluded.
Outcomes	The primary outcome was a composite of progression of AKI, receipt of dialysis, or death within 14 days of randomization or hospital discharge. Progression of AKI was defined by achieving a higher KDIGO AKI stage than the stage present at initial randomization (stage 2 represents a doubling of creatinine, stage 3 a tripling of creatinine). Secondary outcomes included the components of the priumary outcome (at 14 days and during the entire admission), the proportion of patients who did not receive one or more MOIs for which they were enrolled within 24 hours and while AKI was still present, the duration of AKI, and the rate of readmission at 30-days. We considered AKI resolved when the more recent measured creatinine no longer met AKI criteria.
